# Translation and Validation of a Culturally Adapted Arabic Version of the Beverage Intake Questionnaire (BEVQ)

**DOI:** 10.7759/cureus.71710

**Published:** 2024-10-17

**Authors:** Abbas A Alkinani, AJ Rohana, Ruhaya Hasan, Wan Muhamad Amir W Ahmad, Saad Abid Al-badri

**Affiliations:** 1 Department of Community Medicine, Universiti Sains Malaysia, Kubang Kerian, MYS; 2 Department of Community Medicine, School of Medical Sciences, Universiti Sains Malaysia, Kubang Kerian, MYS; 3 School of Dental Sciences, Universiti Sains Malaysia, Kota Bharu, MYS; 4 Department of Internal Medicine, College of Medicine, Wasit University, Wasit, IRQ

**Keywords:** adolescent, arabic translation, beverage intake questionnaire, cultural adaptation, translation, validation

## Abstract

Background: The prevalence of sugar-sweetened beverage (SSB) consumption is on the rise among Arabic adolescents, with a notable increase observed in Iraq. However, no validated tool currently exists to assess SSB consumption apart from the frequency of SSB intake within this population. The objective of this study is to evaluate the beverage consumption patterns of Arabic-speaking adolescents using a validated Beverage Intake Questionnaire (BEVQ) specifically designed for this population.

Methodology: The BEVQ has been authorized by the original author and was meticulously translated through a 10-step protocol. The content validity of the BEVQ was rigorously evaluated by four independent experts using the item-level content validity index (I-CVI), scale-level content validity index average (S-CVI/Ave), sum of the content validity index/universal agreement (S-CVI/UA), and the modified kappa statistic (*κ*_m_). The face validity was also conducted on 30 adolescents, ensuring clarity and comprehensive validation.

Results: The translation process required minor modifications to ensure linguistic and cultural equivalence to the original questionnaire. The Arabic version of the BEVQ (BEVQ-A) achieved S-CVI/Ave scores ranging from 0.90 to 0.98 and S-CVI/UA scores ranging from 0.75 to 1.00. The modified kappa statistic (*κ*_m_) indicated that the majority of items were categorized as good to excellent. These scores confirmed that the BEVQ-A possessed robust content validity. Additionally, the BEVQ-A demonstratedcomprehensive and clear face validity, with a sum of face validity index (S-FVI) score of 0.97.

Conclusion: In conclusion, the Arabic-translated version of the BEVQ is a valid and reliable instrument for assessing total beverage consumption among Arabic-speaking adolescents.

## Introduction

The alarming rise in sugar-sweetened beverage (SSB) consumption among Arabic adolescents has reached critical levels, particularly in Iraq, where 40% of adolescents consume these beverages five to seven times per week [1-3]. This trend has become a significant public health issue with dire implications for future generations, as high consumption of unhealthy beverages is strongly associated with various health problems, including obesity, type 2 diabetes, dental cavities, and metabolic syndrome [4].

A primary source of unhealthy beverages in the Arabic diet is added sugars, with additional syrups and sugars added during food preparation [5]. Adolescents frequently consume SSBs, including soft drinks, fruit drinks with added sugars, energy drinks, sports drinks, sweetened teas, and flavored coffees. In addition, artificially sweetened beverages, such as diet sodas and zero-calorie drinks, along with flavored milk, milkshakes, and juices with added sugars, are also significant sources of sugar intake [6]. While zero-calorie drinks are marketed as healthier alternatives, many contain artificial sweeteners, such as aspartame or sucralose, which have been associated with adverse health effects. Research has shown that these sugar substitutes may disrupt metabolic processes, alter gut microbiota, and increase the risk of glucose intolerance. Furthermore, prolonged consumption of artificial sweeteners may enhance cravings for sweet foods, potentially promoting unhealthy dietary patterns [7]. The World Health Organization recommends that no more than 10% of total daily energy intake should come from free sugars for all age groups [8]. This intake should ideally be reduced to less than 5% of total daily energy intake for additional health benefits. However, children and adolescents in the Eastern Mediterranean region frequently exceed these recommendations, consuming SSBs at levels that contribute significantly more than the recommended percentage of total energy intake [8].

Globally, over 40 countries have implemented taxes on SSBs to combat health issues like obesity and diabetes [9]. Despite this trend, Iraq did not adopt such measures, indicating a potential area for public health policy development to address high sugar consumption among its population [9]. Assessing dietary intake, especially beverage consumption, is vital to evaluate the nutritional status among adolescents and their health implications. Although many studies have validated dietary assessment tools in various linguistic and cultural contexts, most of the literature focused on Western, English-speaking populations. Arabic, the fifth most spoken language with over 450 million native speakers and official status in 22 countries, is underrepresented in this research [10]. An existing tool, i.e., the Beverage Intake Questionnaire (BEVQ), has been developed for the Western culture but there is a lack of adaptation in Arabic cultural and dietary contexts. Consequently, there is a critical need to translate, culturally adapt, and validate the BEVQ for Iraqi adolescents to ensure accurate and reliable measurement of their beverage intake.

Research challenges in questionnaire translation include navigating cultural differences and ensuring representative samples. Additionally, the cross-sectional design may overlook seasonal variations, which affected beverage intake. Despite these limitations, this present study has attempted to translate and validate the BEVQ to tailor similar SSB intake among Arabic-speaking populations, in particular adolescents. In achieving a valid questionnaire, the process must include precise translation steps and cultural adaptation supported by evidence of cross-language and cross-cultural equivalence [11], and rigorous validation assessment by experts [12,13]. This process should involve face validity to ensure the content is comprehensive and the items are clear [12].

The BEVQ is a validated 12-item tool used to estimate habitual beverage intake over the past month across 12 beverage categories in American children and adolescents [14]. This brief assessment tool provides an estimate of total beverage and SSB intake, along with milk, water, and other drinks. This study aims to translate, culturally adapt, and validate the BEVQ into Arabic to ensure its efficacy in assessing beverage intake among Iraqi adolescents aged 16 to 18 years.

## Materials and methods

The cultural translation and adaptation of the Beverage Intake Questionnaire (BEVQ)

The BEVQ assesses beverage consumption over the past month. Developed by Hill et al., this instrument evaluates children and adolescents across 12 items related to their consumption of the total beverage, SSB intake, and milk quantity (fl oz, kcal, gram) [14]. The development of the original BEVQ included the assessment of the study, which assessed the validity and reliability of the BEVQ through four laboratory sessions over two to three weeks, using a randomized visit sequence to avoid influencing dietary habits. Participants completed the BEVQ at different visits depending on their sequence and received a food-recording booklet for reference during the 24-hour dietary recalls (24HR). The 24HRs were conducted on non-consecutive days, including one weekend day and three weekdays. The results showed acceptable test-retest reliability, with R-values ranging from 0.3 to 0.949, and no significant differences between the BEVQ-1 and BEVQ-2 responses, except for total beverages. These findings indicated that the BEVQ was a valid and reliable tool for assessing habitual water and total SSB intake in children and adolescents [14]. In the present study, permission was obtained from the author to translate and validate the BEVQ questionnaire to be utilized among Iraqi adolescents.

Prior to the translation process, a review based on previous literature was conducted. The translation process was implemented using the 10 steps in the guidelines by Wild et al. [15]. In the preparation step, permission was granted to the original developer of BEVQ. During the forward translation step, two native Iraqis proficient in English and Arabic independently translated the questionnaires, which were later reviewed, and the discrepancies between the two languages mentioned above were discussed. The discussion was also conducted to find the differences between both translators before summarizing the final decision. In the reconciliation step, a panel reviewed and reconciled discrepancies to ensure cultural appropriateness, crafting a standardized forward translation. The backward translation step involved independent translators, confirming conceptual, item, and semantic equivalence. The expert team conducted the backward translation review step to ensure translation equivalence. In the harmonization step, differences were resolved to match the original instrument, establishing a pre-final version. The cognitive debriefing step involved testing the pre-final version with 10 secondary school students (n = 30) and their respective parents and integrating their feedback. In the review of cognitive debriefing results and finalization step, inconsistencies were addressed based on the feedback provided. The proofreading step ensured accuracy and consistency in the final translation. Finally, the production of the final Arabic version step involved preparing a comprehensive translation report. This systematic approach was purposely used to ensure the validity and reliability of the translated BEVQ for cross-cultural research.

Validating the Arabic version of the Beverage Intake Questionnaire (BEVQ-A)

Content Validity (Linguistic Validation)

In April 2023, a panel of four highly qualified specialists from Iraq, proficient in both Arabic and English, was appointed to commence the content assessment process. The expert panel conducted a thorough evaluation of the Arabic version of the Beverage Intake Questionnaire (BEVQ-A) to ensure its relevance and accuracy for the target population [16,17]. The expert committee consisted of two nutritionists and two linguists. One nutritionist holds a PhD from the University of Putra Malaysia, while the other is currently pursuing a PhD at the University of Science Malaysia. Among the linguists, one holds a PhD from the University of Technology Malaysia (UTM) and serves as a lecturer in the Department of Translation at the College of Arts, Wasit University, Iraq. The other linguist has a Master of Science degree in linguistics and translation from the University of Putra Malaysia. All members were fluent in English and native Arabic speakers. The panel reviewed the wording and content of the questionnaire to ensure its cultural appropriateness and relevance for the Iraqi population. Additionally, they evaluated each item based on four key criteria: relevance, clarity, simplicity, and potential ambiguity.

Using quantitative content validity methods, experts assessed the item-level content validity index (I-CVI), scale-level content validity index-average (S-CVI/Ave), the sum of the content validity index/universal agreement (S-CVI/UA), and modified kappa agreement. These measures evaluate inter-rater agreement among experts. Consensus was reached on the relevance, clarity, simplicity, and lack of ambiguity of all items in the BEVQ. Experts rated items on a four-point scale (1 = not relevant, 2 = needs revision, 3 = relevant but needs minor revision, and 4 = very relevant). Items rated 1 or 2 were considered invalid, while those rated 3 or 4 were considered valid [18]. The I-CVI measures the agreement on each item, with values ranging from 0 to 1, while S-CVI measures the proportion of items rated as content valid. The S-CVI/UA indicates the proportion of items that received unanimous agreement. The kappa statistic adjusted for chance agreement, providing a measure of inter-rater reliability. An item was approved if I-CVI ≥ 0.78 [16-17]. Items with I-CVI ≥ 0.79 were appropriate, those between 0.70 and 0.79 needed revision, and those ≤0.70 were removed. An S-CVI/Ave of ≥0.90 indicated acceptable content validity [17,19]. S-CVI/UA values of 0.70 portrayed that experts unanimously agreed upon 70% of the items [17,19]. Calculations used in the study for I-CVI, S-CVI/Ave, and S-CVI/UA were as follows:

$I-CVI =(n(3 or 4))/N$

Where *I-CVI* is the item content validity index, *n(3 or 4)*​ is the number of experts rating the item as 3 or 4, and *N* is the total number of experts [17].

$SCVI/Ave = (\Sigma I-CVI) / N$

Where *S* represents scale, *CVI* is the content validity index, *Σ* represents sum, and *N* is the total number of items [20].

$𝑆&minus;𝐶𝑉𝐼/𝑈𝐴 = 𝑚 / 𝑁$

Where *S* represents scale, *CVI* is the content validity index, *UA* is the universal agreement, *m* is the number of items with unanimous agreement, and *N* is the total number of items [20].

According to Viera et al., a kappa value below 0 signified less than chance agreement, a range of 0.01 to 0.20 suggested slight agreement, 0.21 to 0.40 indicated fair agreement, 0.41 to 0.60 signified moderate agreement, 0.61 to 0.80 indicated good agreement, and 0.81 to 0.99 indicated nearly perfect agreement [21].

$𝑃𝑐 = (𝑁! / (𝐴! &lowast; (𝑁 &minus; 𝐴)!)) &lowast; 5^𝑁$

Where *Pc* ​is the probability of chance agreement, *N* is the number of experts, *A* is the number of items agreed upon, *!* represents factorial, and *5* is the number of rating categories [22].

$Kappa = (I-CVI &minus; 𝑃𝑐) / (1 - 𝑃𝑐)$

Where *K* is the kappa, *I* is the item, *CVI* is the content validity index, and *Pc *is the probability of chance agreement [21].

Face Validity

Face validity assesses the extent to which test items appear to accurately reflect the construct as defined by the evaluation of researchers. After confirming content validity, response process validity was also examined [12]. Face validity further considered whether evaluators found the assessment items relevant to the intended construct and research objectives.

The assessment tool was designed to target both adolescents and their parents. Although 10 raters (n = 10) were typically the minimum for response process validation [23], most studies involved 30 or more raters [24]. In this study, face-to-face validation involved 30 students who were randomly selected from four schools in Kut City. Research indicated that 30 raters provide the most robust face validity results [13]. Feedback from raters, which had been gathered verbally and in writing, was used to refine the clarity of the items. Each item received a rating from 1 to 4, where 3 or 4 signified clarity and 1 or 2 indicated lack of clarity.

To ensure accuracy, a face validity index (FVI) score of at least 0.80 is required [13,24]. The FVI was calculated using these formulas: I-FVI = (number of agreed items)/(total number of raters); S-FVI/Ave = (sum of I-FVI scores)/(number of items) [13].

Ethical consideration

Ethical approval for this study was granted by the Human Research Ethics Committee at Universiti Sains Malaysia (USM) (Protocol code: USM/JEPeM/KK/23030276). Additionally, ethical clearance for the participation of human subjects was obtained from the Branch of Studies and Research/Section of Training and Preparation under the Director General of Wasit Education (Ref.: 71654; Date: November 28, 2022).

## Results

Cultural translation and adaptation of the BEVQ-A

The translation and cultural adaptation of the BEVQ from English to Arabic (Iraq) involved meticulous modifications to ensure semantic, content, technical, criterion, and construct-concept equivalence (Appendix). Semantic equivalence preserved the original meaning for cultural recognition. Content equivalence ensured relevance by including commonly available traditional drinks such as tamarind juice and sherbet. Technical equivalence involved adapting units and product names by using milliliters alongside fluid ounces and maintaining the color coding for milk products. Criterion equivalence was maintained by translating popular beverage brands into local equivalents, such as *Tiger* and *Power Horse*. Construct-concept equivalence maintains the understanding of underlying concepts, such as the addition of milk or creamer to tea or coffee. This comprehensive approach ensures the BEVQ-A remains valid across cultural contexts.

Content validity of the BEVQ-A

The content validity of items in the Arabic version of the BEVQ was assessed by four experts, resulting in comprehensive data on various metrics. For the criterion of relevance, the expert consensus (EC) scores ranged from 3 to 4 across the items, with a mean item content validity index (I-CVI) of 0.94 and a universal agreement (S-CVI/UA) ranging from 0.75 to 1.00. The modified kappa (κm) values were high, indicating strong agreement among the experts, with values of 1.00 for most items, except for Q1, Q3, and Q6, which had *κ_m_* values of 0.67, as illustrated in Table 1.

**Table 1:** Expert agreement (n = 4) on the content validity of items in the Arabic version of the BEVQ. BEVQ = Beverage Intake Questionnaire; CVM = content validity metrics; EC = expert consensus; I-CVI = item content validity index; Rc = probability of chance occurrence; κm = modified kappa agreement; S-CVI/Ave = sum of the content validity index/average (number of items); S-CVI/UA = sum of the content validity index/universal agreement (lower, upper); INTP = interpretation; App. = approved; Rev. = need revision; Exc. = excellent; Gd. = good; * = poor.

Criteria: CVM	Item												S-CVI/Ave	S-CVI/UA
	Q1	Q2	Q3	Q4	Q5	Q6	Q7	Q8	Q9	Q10	Q11	Q12		
Relevance: EC	3	4	3	4	4	3	4	4	4	4	4	4	0.94	0.75, 1.00
I-CVI	0.75	1	0.75	1	1	0.75	1	1	1	1	1	1		
Rc	0.25	0.06	0.25	0.06	0.06	0.25	0.06	0.06	0.06	0.06	0.06	0.06		
κ_m_	0.67	1.00	0.67	1.00	1.00	0.67	1.00	1.00	1.00	1.00	1.00	1.00		
Clarity: EC	2	4	3	4	4	3	3	4	4	4	4	4	0.90	0.75, 1.00
I-CVI	0.5	1	0.75	1	1	0.75	0.75	1	1	1	1	1		
Rc	0.38	0.06	0.25	0.06	0.06	0.25	0.25	0.06	0.06	0.06	0.06	0.06		
κ_m_	0.20*	1.00	0.67	1.00	1.00	0.67	0.67	1.00	1.00	1.00	1.00	1.00		
Simplicity: EC	3	4	4	4	4	4	4	4	4	4	4	4	0.98	0.92, 1.00
I-CVI	0.75	1	1	1	1	1	1	1	1	1	1	1		
Rc	0.25	0.06	0.06	0.06	0.06	0.06	0.06	0.06	0.06	0.06	0.06	0.06		
κ_m_	0.67	1.00	1.00	1.00	1.00	1.00	1.00	1.00	1.00	1.00	1.00	1.00		
Ambiguity: EC	2	4	4	4	4	4	4	4	4	4	4	4	0.96	0.92, 1.00
I-CVI	0.5	1	1	1	1	1	1	1	1	1	1	1		
Rc	0.38	0.06	0.06	0.06	0.06	0.06	0.06	0.06	0.06	0.06	0.06	0.06		
κ_m_	0.20*	1.00	1.00	1.00	1.00	1.00	1.00	1.00	1.00	1.00	1.00	1.00		
INTP: I-CVI	Rev.	App.	Rev.	App.	App.	Rev.	Rev.	App.	App.	App.	App.	App.		
κ_m_	Gd.	Exc.	Gd.	Exc.	Exc.	Gd.	Gd.	Exc.	Exc.	Exc.	Exc.	Exc.		

Regarding clarity, the EC scores varied from 2 to 4, with an overall S-CVI/Ave of 0.90 and an S-CVI/UA between 0.75 and 1.00. The I-CVI values ranged from 0.5 to 1.00, with item Q1 receiving the lowest I-CVI (0.50) and modified kappa agreement (0.20), suggesting it might need revision. The probability of chance occurrence was generally low, further supporting the validity of these evaluations.

For simplicity, the EC ratings were consistently high (3 or 4), resulting in an S-CVI/Ave of 0.98 and an S-CVI/UA between 0.92 and 1.00. All items had an I-CVI of 1.00, except for Q1 and Q6, which had an I-CVI of 0.75. The κm values were uniformly 1.00, except for Q1, which had a κm of 0.67, indicating excellent agreement among experts.

The ambiguity criterion also showed high EC scores, with an S-CVI/Ave of 0.96 and an S-CVI/UA ranging from 0.92 to 1.00. Most items achieved an I-CVI of 1.00, with only Q1 having a lower I-CVI of 0.5 and a corresponding κm of 0.20, suggesting it may require additional refinement based on expert feedback.

In summary, the expert agreement on the content validity of the Arabic BEVQ items is generally satisfied across all criteria. Items identified with lower modified kappa agreement and I-CVI values, particularly in relevance and clarity, should be reviewed for potential revisions to enhance their validity. The high overall S-CVI/Ave and S-CVI/UA values support the robustness of the Arabic BEVQ, ensuring its content validity is maintained through expert consensus.

Face validity of the BEVQ-A

Table 2 presents the average item face validity index (I-FVI) of the BEVQ-A, evaluated by 30 raters. The I-FVI indicated the proportion of raters who agreed on the face validity of each item. For the majority of the items (Q2 to Q5, Q7 to Q10, and Q12), a perfect agreement was achieved, with all 30 raters in consensus, resulting in an I-FVI of 1.00. Items Q1 and Q6 had a slightly lower agreement with 27 raters in consensus, corresponding to an I-FVI of 0.90. Item Q11 had the lowest agreement among the raters, with 25 in agreement and an I-FVI of 0.83.

**Table 2:** The average item for the face validity index of BEVQ-A from 30 raters. BEVQ-A = Arabic version of the Beverage Intake Questionnaire; I-FVI = item face validity index; S-FVI/Ave = sum of the face validity index/average (number of items); INTP = interpretation; App. = approved.

Item	I-FVI	INTP
Q1, Q6	0.90	App.
Q2, Q3, Q4, Q5, Q7, Q8, Q9, Q10, Q12	1.00	App.
Q11	0.83	App.
S-FVI/Ave	0.97	

The overall face validity of the BEVQ-A items is high, as reflected by the sum of the face validity index/average (S-FVI/Ave) of 0.97. All items were interpreted as approved, indicating that they were deemed face-valid by the majority of the raters. This high level of agreement suggested that the items of the BEVQ-A were clear and appropriately captured the constructs they were intended to measure, confirming the instrument's face validity.

## Discussion

This study aims to translate, culturally adapt, and validate the Arabic-language version of the BEVQ for use among Iraqis. Through a rigorous process of translation and cultural adaptation, the Arabic BEVQ achieved semantic, content, technical, criterion, and construct-concept equivalence in relation to the original English version. Semantic equivalence was preserved by maintaining the meaning of the original text, while content equivalence was ensured by including locally consumed beverages. Technical equivalence was achieved by adapting units and product names, and criterion equivalence by incorporating local brands. Construct-concept equivalence confirmed that the core concepts of the questionnaire were understood similarly across cultures. The expert evaluation demonstrated high content validity, with only a few items, such as Q1 and Q6, requiring revisions due to lower modification kappa agreement values. The face validity results were also strong, with most items achieving perfect agreement among raters. Overall, this comprehensive validation process ensures the reliability and cultural relevance of the Arabic BEVQ for cross-cultural research on beverage intake.

The process of translating and culturally adapting the BEVQ from English to Arabic (Iraq) effectively ensured semantic, content, technical, criterion, and construct-concept equivalence. These findings align with existing literature on questionnaire adaptation, which emphasizes the importance of maintaining these forms of equivalence [11]. The high content validity and face validity achieved in this study are consistent with previous studies that report similar validity indices for well-validated instruments [12,13]. Differences in specific items, like Q1 and Q6, suggest potential cultural nuances or translation ambiguities, which have also been noted in other dietary assessment adaptations [25]. By including culturally familiar drinks and local brands, this study reinforces the importance of cultural relevance, a key factor in accurate dietary assessments. The validated Arabic BEVQ provides a reliable tool for cross-cultural research and public health interventions in Iraq, contributing significantly to the field by facilitating accurate beverage consumption assessment in Arabic-speaking populations. Future studies could focus on refining problematic items and exploring regional differences to further enhance the tool's applicability.

Two studies have addressed the development and translation of beverage intake questionnaires for the Saudi Arabian population. The first study [26] had several limitations. The questionnaire, adapted from prior research, lacked a comprehensive content validity process, which may have compromised its cultural relevance. The study was based on a small sample of adults, limiting the generalizability of the results. Moreover, it did not account for total beverage intake, SSBs, or milk consumption based on caloric intake, restricting the scope of the findings. The focus on a specific age group further constrained the ability to apply the results to the broader population. Future research should employ larger, more diverse samples and include validated beverage categories that account for caloric intake. The second study [27], which involved translating an existing questionnaire, also faced significant limitations. Notably, the translation process lacked a formal validation procedure to ensure semantic, content, technical, criterion, and construct equivalence. This absence raised concerns about the consistency and reliability of the translated items. Additionally, no expert-driven content validation index was utilized to confirm the relevance and appropriateness of the questionnaire items for the target population. Without these crucial validation steps, the accuracy and cultural adaptability of the translated questionnaire in assessing beverage consumption remains uncertain.

The study on the BEVQ faced significant limitations in cultural translation and adaptation, as well as in maintaining content and face validity. Despite efforts to ensure semantic, content, technical, criterion, and construct-concept equivalence, challenges arose due to distinct differences in beverage consumption patterns between Western and Iraqi cultures. For instance, American students exhibited a diverse range of beverage preferences, including water, SSBs, milk, and fruit juice [28]. In contrast, Iraqis predominantly consume traditional beverages such as tea and coffee, alongside a growing trend toward bottled water and soft drinks [29]. This study required careful adaptation of culturally relevant drinks, such as tamarind juice, necessitating expert consultation, and validation through face validity [15]. Content validity, assessed by four experts, was generally high but showed lower agreement for items like Q1 and Q6, indicating the need for further refinement [17]. Face validity, although achieving a high overall score, highlighted the difficulty of ensuring all translated items were clear and culturally appropriate, reflecting the subjective nature of this validation method. Face validity, although achieving a high overall score, highlighted the difficulty of ensuring all translated items were clear and culturally appropriate, reflecting the subjective nature of this validation method. Face validity primarily relies on the judgment of experts and participants to assess whether the instrument appears to measure what it is intended to measure [13,18]. This method is inherently subjective, as it depends on individual perceptions and cultural contexts, which can vary widely. As a result, while face validity can provide initial insights into the appropriateness of the translated items, it may not fully capture subtle cultural nuances, leading to potential misunderstandings or misinterpretations [18]. Consequently, achieving a high score in face validity does not necessarily guarantee that all items are perfectly clear and culturally appropriate, underscoring the importance of complementing face validity with other more objective validation methods. Overall, while the BEVQ was successfully translated and culturally adapted, these limitations underscore the complexities of cross-cultural research and the necessity for continuous refinement to maintain reliability and cultural relevance [13].

The findings on the cultural adaptation and validity of the Arabic version of the BEVQ offer valuable practical applications, particularly in assessing mean beverage intake and calorie consumption from SSBs and milk among Arabic-speaking adolescents, crucial for addressing obesity linked to high beverage consumption. Future research should expand on these results by exploring additional factors influencing beverage intake, refining the BEVQ for accuracy, and incorporating technological advancements to improve data collection. This research should align with recent studies on beverage intake and obesity, aiming to inform effective public health strategies [30].

Finally, the study successfully translated and culturally adapted the BEVQ from English to Arabic for Iraqi adolescents, ensuring semantic, content, technical, criterion, and construct equivalence. The Arabic BEVQ demonstrated content and face validity, though some items need further refinement. It is now a reliable tool for cross-cultural research and public health interventions in Iraq, aiding in beverage consumption assessments. However, ongoing refinement is necessary to address cultural nuances and improve its reliability for future research.

## Conclusions

The BEVQ-A has been successfully translated and validated as a reliable instrument for assessing total average beverage, SSBs, and milk consumption among Iraqi adolescents in Wasit. Experts affirmed strong validity and face content validity across most beverage categories, with only minimal modifications observed in certain items. Modifications were implemented to ensure that the semantic meaning, content relevance, technical details, measurement criteria, and underlying concepts were preserved and culturally adapted. Utilizing this Arabic version of the validated tool would allow researchers to gain valuable insights into beverage consumption behaviors. Furthermore, it will assist in developing more effective early intervention programs aimed at improving dietary assessment-related outcomes in this age group.

## Appendices

**Table 3:** Translation and back translation of the BEVQ. Note: O = original English version item; A = Arabic version item; B = back-translated English version item; ^a^ = translator modification; ^b^ = experts’ modification; ^c^ = adolescent modification.

Item No.	Beverage Intake Questionnaire (BEVQ)
01	(O) Water or unsweetened sparkling water
	(A) الماء
	(B) Water ^(b & c)^
02	(O) 100% fruit juice
	(A) عصير فواكه طازج 100%
	(B) Fresh fruit juice 100% ^(a)^
03	(O) Sweetened juice, beverage/drink (fruit punch, juice cocktail, Sunny Delight, Capri Sun)
	(A) مشروب عصير محلى / مشروب محلى: شربت، عصير تمر هندي، وشربت الزبيب.
	(B) Sweetened juice beverage/local drink: sherbet, tamarind juice, Shirbat Zabib) ^(b & c)^
04	(O) Whole milk: red cap, reduced fat milk 2%: purple cap or chocolate milk
	(A) الحليب كامل الدسم: غطاء أحمر ، حليب قليل الدسم 2٪: غطاء أرجواني ، أو حليب شوكولاتة.
	(B) Whole milk: red cap, low-fat milk 2%: purple cap or chocolate milk
05	(O) Low fat 1%: green cap, fat-free/skim milk: light blue cap, buttermilk or soy milk
	(A) قليل الدسم 1٪: غطاء أخضر ، حليب خالي من الدسم / خالي الدسم: كوب أزرق فاتح ، لبن أو حليب الصويا.
	(B) Low-fat 1%: green cap, fat-free/skim milk: light blue cup, yogurt, or soy milk ^(a)^
06	(O) Nut milk (almond, cashew, coconut), flavored, original, or plain unsweetened
	(A) حليب الجوز (اللوز ، الكاجو ، جوز الهند) منكه أو أصلي أو عادي غير محلى
	(B) Nut milk (almond, cashew, coconut), flavored, original, or plain unsweetened
07	(O) Soft drinks, regular
	(A) المشروبات الغازية العادية
	(B) Regular soft drinks ^(a)^
08	(O) Energy & sports drinks, regular (Red Bull, Gatorade, Powerade)
	(A) مشروبات الطاقة والرياضة، العادية (ريد بُل، تايجر، باور هورس، بلو باور).
	(B) Regular energy & sports drinks (Red Bull, TIGER, Power Horse, Blue Power) ^(b & c)^
09	(O) Diet or artificially sweetened soft drinks, energy & sports drinks (Diet Coke, Crystal Light, artificially sweetened sparkling water, Sugar-Free or Total Zero Red Bull, Powerade Zero)
	(A) الدايت أو المشروبات الغازية المحلاة صناعيا، ومشروبات الطاقة والرياضة (بيبسي دايت، ريد بُل زيرو سكر، باور هورس زيرو سكر).
	(B) Diet or artificially sweetened carbonated drinks, energy and sports drinks) Pepsi Diet, Red Bull Zero Sugar, Power Horse Zero Sugar) ^(b & c)^
10	(O) Sweet tea (with sugar)
	(A) شاي حلو (بالسكر)
	(B) Sweet tea (with sugar)
11	(O) Tea or coffee, black (no creamer or milk), sugar, artificial sweetener, N/A
	(A) شاي أو قهوة سادة (بدون مبيض أو حليب) السكر التحلية الاصطناعية غير متاح
	(B) Black tea or coffee (no creamer or milk), sugar, artificial sweetener, N/A
12	(O) Tea or coffee (with milk &/or creamer), sugar, artificial sweetener, N/A, milk &/or creamer, milk half & half cream, N/A, creamer: Flav. plain, sugar-free
	(A) شاي أو قهوة (مع حليب و / أو مبيض) السكر التحلية الاصطناعية غير متاح حليب و / أو مبيض الحليب نصف ونصف أو كريم غير متاح كريمر: منكة سادة خالي من السكر
	(B) Tea or coffee (with milk &/or creamer), sugar, artificial sweetener, N/A, milk &/or creamer, milk half & half cream, N/A, creamer: Flav. plain, sugar-free

## References

[1] Selman NA, Hussain AMA, Mera NAA (2024). The prevalence of nutritional status and obesity in adolescents in Babylon Province, Iraq. Med J Babylon.

[2] Alasqah I, Mahmud I, East L, Alqarawi N, Usher K (2021). Dietary behavior of adolescents in the Qassim region, Saudi Arabia: a comparison between cities with and without the Healthy Cities Program. Int J Environ Res Public Health.

[3] Boulhannaa A, Anarghoub H, Ihbourc S, Najimi M, Chigr F (2021). Prevalence of physical activity, sedentary behaviour and unhealthy dietary habits among Moroccan adolescents. Turkish J Comput Math Educ.

[4] Neelakantan N, Park SH, Chen GC, van Dam RM (2021). Sugar-sweetened beverage consumption, weight gain, and risk of type 2 diabetes and cardiovascular diseases in Asia: a systematic review. Nutr Rev.

[5] Mumena WA, Kutbi HA (2024). Sources of free sugar in the diet of Saudi children. Front Public Health.

[6] Snetselaar LG, de Jesus JM, DeSilva DM, Stoody EE (2021). Dietary guidelines for Americans, 2020-2025: understanding the scientific process, guidelines, and key recommendations. Nutr Today.

[7] Szyca M, Wawrzeńczyk M, Jędrzejewska B, Przeradzki J, Kotulska M, Borycka A, Lichman M (2023). Artificial sweeteners and their impact on human health. Med Og Nauk Zdr.

[8] (2024). WHO. Reducing consumption of sugar-sweetened beverages to reduce the risk of childhood overweight and obesity. World Health Organization.

[9] World Health Organization (2023). Global Report on the Use of Sugar-Sweetened Beverage Taxes, 2023. use of sugar-sweetened beverage taxes.

[10] (2024). Istizada. Complete list of Arabic speaking countries - 2023 update. https://istizada.com/complete-list-of-arabic-speaking-countries/.

[11] Jomori MM, Caraher M, Bernardo GL, Uggioni PL, Echevarria-Guanilo ME, Condrasky M, Proença RP (2021). How was the cooking skills and healthy eating evaluation questionnaire culturally adapted to Brazil?. Cien Saude Colet.

[12] Yusoff MSB (2019). ABC of content validation and content validity index calculation. Educ Med J.

[13] Yusoff MSB (2019). ABC of response process validation and face validity index calculation. Educ Med J.

[14] Hill CE, MacDougall CR, Riebl SK, Savla J, Hedrick VE, Davy BM (2017). Evaluation of the relative validity and test-retest reliability of a 15-item beverage intake questionnaire in children and adolescents. J Acad Nutr Diet.

[15] Wild D, Grove A, Martin M, Eremenco S, McElroy S, Verjee-Lorenz A, Erikson P (2005). Principles of good practice for the translation and cultural adaptation process for patient-reported outcomes (PRO) measures: report of the ISPOR Task Force for translation and cultural adaptation. Value Health.

[16] Lynn MR (1986). Determination and quantification of content validity. Nurs Res.

[17] Polit DF, Beck CT (2006). The content validity index: are you sure you know what's being reported? Critique and recommendations. Res Nurs Health.

[18] Amir W and Mohamad N (2021). How to develop and validate a questionnaire?. Malaysia: Penerbit Universiti Sains Malaysia.

[19] Polit DF, Beck CT, Owen SV (2007). Is the CVI an acceptable indicator of content validity? Appraisal and recommendations. Res Nurs Health.

[20] DeVon HA, Block ME, Moyle-Wright P (2007). A psychometric toolbox for testing validity and reliability. J Nurs Scholarsh.

[21] Viera AJ, Garrett JM (2005). Understanding interobserver agreement: the kappa statistic. Fam Med.

[22] DeVellis RF, Thorpe CT (2022). Scale Development: Theory and Applications. Fifth Edition. SAGE Publications.

[23] Rahim AFA, Yusof MSB (2016). Validity evidence of a multiple mini interview for selection of medical students: Universiti Sains Malaysia experience. Educ Med J.

[24] Lau A, Yusof MSB, Lee YY (2017). Development, translation and validation of questionnaires for diarrhoea and respiratory-related illnesses during probiotic administration in children. Educ Med J.

[25] Livingstone KM, Love P, Mathers JC, Kirkpatrick SI, Olstad DL (2023). Cultural adaptations and tailoring of public health nutrition interventions in Indigenous peoples and ethnic minority groups: opportunities for personalised and precision nutrition. Proc Nutr Soc.

[26] Aldhirgham TM, Almutairi LA, Alraqea AS, Alqahtani AS (2023). Online Arabic Beverage Frequency Questionnaire (ABFQ): evaluation of validity and reliability. Nutr J.

[27] Islam MA, Al-karasneh AF, Hussain AB (2020). Assessment of beverage consumption by young adults in Saudi Arabia. Saudi Pharm J.

[28] Miller G, Merlo C, Demissie Z, Sliwa S, Park S (2017). Trends in beverage consumption among high school students - United States, 2007-2015. MMWR Morb Mortal Wkly Rep.

[29] Hassan HI Sr, Othman SM (2024). Sugar-sweetened beverage consumption and its association with dental caries among adolescents in Erbil, Iraq: a cross-sectional study. Cureus.

[30] Alkinani AAA, Jalil R, Hasan R, Mohamed WMIW (2022). The relationship between sugar-sweetened beverages and obesity between children and adolescents in Eastern Mediterranean region : systematic review. The 1st International & 14th National Conference on Innovations in Health.

